# Meta regression of endoscopic sleeve gastroplasty versus intragastric balloon investigating influence of duration and baseline body mass index

**DOI:** 10.1038/s41598-026-38374-1

**Published:** 2026-02-04

**Authors:** Po-Feng Huang, Hsuan-Wei Chen, Tien-Yu Huang, Peng-Jen Chen, Chi-Wei Yang

**Affiliations:** 1 Division of Gastroenterology, Department of Internal Medicine, Kaohsiung Armed Forces General Hospital, Kaohsiung, Taiwan, ROC; 2 Division of Gastroenterology, Department of Internal Medicine, Tri-Service General Hospital, National Defense Medical University, No.325, Sec.2, Chenggong Rd., Neihu District, Taipei City, 114202 Taiwan, ROC

**Keywords:** Endoscopic sleeve gastroplasty, Intragastric balloon, Obesity, Weight loss, Meta-regression, Meta-analysis, Diseases, Gastroenterology, Medical research

## Abstract

Endoscopic sleeve gastroplasty (ESG) and intragastric balloon (IGB) are established endoscopic bariatric therapies (EBTs) for obesity, but their comparative efficacy and safety remain uncertain. We conducted a systematic review and meta-analysis of comparative studies evaluating ESG versus IGB. The primary outcome was percentage of total body weight loss (%TBWL), assessed at the longest available follow-up and stratified timepoints (1, 3, 6, and 12 months). To explore temporal trends, we performed a meta-regression using follow-up duration as a continuous moderator. Secondary outcomes included a meta-regression based on baseline BMI differences and a pooled analysis of adverse events. Six comparative studies (n = 5330) were included. ESG was associated with significantly greater %TBWL than IGB (mean difference: 2.541; 95% CI 0.754 to 4.327; *p* = 0.005), with increasing benefit over time. Meta-regression confirmed a significant positive association between follow-up duration and treatment effect (*p* = 0.0006). A separate meta-regression revealed greater efficacy of ESG in patients with higher baseline BMI (*p* = 0.0001). Overall adverse event rates were comparable between the two groups (*p* = 0.20) ; however, IGB showed observed trends toward higher device intolerance and early removal rates in individual cohorts. Our findings suggest that ESG may offer a therapeutic advantage over IGB regarding weight loss efficacy, a trend that appears more pronounced in cohorts with higher obesity severity and across extended follow-up periods. ESG offers a comparable safety profile to IGB and may represent a more durable endoscopic bariatric option with potential advantages in procedural tolerability. Long-term trials are needed to validate its metabolic and clinical advantages beyond 12 months.

**Supplementary Information:**

The online version contains supplementary material available at 10.1038/s41598-026-38374-1.

## Introduction

Obesity has emerged as one of the most urgent global health threats of the twenty-first century, with over 1 billion people living with obesity in 2022—accounting for 13% of the world’s population. By 2050, projections indicate that more than 3.8 billion adults—over half of the global adult population—will be overweight or obese^1^. This dramatic rise underscores the pressing need for effective, durable, and minimally invasive weight loss interventions. Among endoscopic bariatric therapies (EBTs), endoscopic sleeve gastroplasty (ESG) and intragastric balloon (IGB) have emerged as promising non-surgical options^2^.

IGB, a temporary endoscopic device designed to induce satiety by occupying gastric space, has been widely adopted for short-term weight loss. While it can result in significant initial reductions in body weight, its benefits often diminish following balloon removal. In a recent study, up to 76% of patients experienced weight regain within three months, averaging a regain of 58% of the initial weight lost^3^. Additionally, intolerance-related adverse effects are common—meta-analytic data indicate that nausea occurs in over 60% and vomiting in more than 50% of patients post-IGB placement, particularly with fluid-filled balloon systems like ORBERA^4^. In contrast, ESG utilizes full-thickness endoscopic suturing with the OverStitch™ system to reduce gastric volume while preserving anatomy^5^. Mimicking the restrictive effects of surgical sleeve gastrectomy, ESG offers lower invasiveness and has shown consistent short- to mid-term efficacy, with pooled TBWL of 15.66–17.56% at 6–12 months and sustained outcomes up to 5 years. Serious adverse events remain low at approximately 1.25%^6^. Furthermore, ESG not only mimics the restrictive effect of surgical sleeve gastrectomy but also offers more durable anatomical changes, potentially leading to sustained weight loss and metabolic benefits^7^.

Previous studies have demonstrated the efficacy of both ESG and IGB, but head-to-head comparisons remain limited, with varying follow-up durations and inconsistent outcome reporting^8,9^. While ESG may offer greater long-term weight loss and tolerability, the extent to which it outperforms IGB—and in which patient populations or timeframes—remains unclear.

In this systematic review and meta-analysis, we aimed to compare the efficacy and safety of ESG versus IGB, with a specific focus on (1) the trajectory of weight loss over time, (2) the influence of baseline body mass index (BMI) differences, and (3) the incidence of adverse events. By incorporating stratified timepoint analyses and meta-regression models, we sought to provide a more individualized and dynamic understanding of how these two EBTs perform across varying follow-up periods and patient characteristics.

## Materials and methods

### Protocol and registration

This meta-analysis was conducted in accordance with the PRISMA 2020 guidelines (see Supplementary Table 1)^10^. As no human subjects were directly involved, approval from an institutional review board and informed consent were not required. The protocol was registered in the INPLASY database (Registration number: INPLASY202580023, 10.37766/inplasy2025.8.0023).

### Search strategy and data collection

Two independent reviewers (P.-F.H. and C.-W.Y.) systematically searched electronic databases including PubMed, Embase, ClinicalKey, Cochrane CENTRAL, ProQuest, ScienceDirect, and Web of Science using the following (‘Endoscopic sleeve gastroplasty’ OR ‘ESG’ OR ‘Endoluminal gastroplasty’ OR ‘Apollo Overstitch’) AND (‘Intragastric balloon’ OR ‘IGB’ OR ‘Gastric balloon’ OR ‘Orbera’ OR ‘ReShape’ OR ‘Spatz’ OR ‘Allurion’). To ensure maximum retrieval, no mandatory outcome terms (e.g., BMI or follow-up) were applied to the core query. The search encompassed all available records up to June 30, 2025. The search encompassed all available records up to June 30, 2025. To capture gray literature and unpublished studies, a supplementary search was also conducted on ClinicalTrials.gov. The comprehensive search strategy is detailed in Supplementary Table S2.

Initially, the same two reviewers screened titles and abstracts to assess eligibility based on consensus. To broaden the scope of inclusion, reference lists of relevant review articles were examined, and additional manual searches were performed^11–16^. Discrepancies between the two reviewers were resolved through consultation with a third reviewer (T.-Y.H.). No language restrictions were applied during the selection process.

### Eligibility criteria and outcomes

This meta-analysis followed the PICO framework (Population, Intervention, Comparison, Outcome), defined as follows: Population—adults with obesity undergoing endoscopic bariatric therapy; Intervention—ESG; Comparison—IGB; Outcomes—body weight reduction measured as percentage of total body weight loss (%TBWL) at various follow-up intervals.

Studies were included if they met the following criteria: (1) randomized or comparative cohort studies involving human participants; (2) studies comparing ESG with IGB for the treatment of obesity; and (3) trials reporting quantitative data on weight-related outcomes (e.g., BMI reduction).

Exclusion criteria included: (1) single-arm or non-comparative designs; (2) lack of extractable data for meta-analysis; (3) overlapping or duplicate study populations; and (4) conference abstracts or non–peer-reviewed reports.

### Risk of bias and evidence quality

The quality of included studies was assessed using the Newcastle–Ottawa Scale (NOS) (Table 2), which evaluates observational studies across three domains: selection of study groups, comparability of groups, and ascertainment of outcomes^17^. Each study was independently reviewed by two investigators (P.-F.H. and C.-W.Y.), with any disagreements resolved through discussion or consultation with a third reviewer (T.-Y.H.). To improve interpretability, each domain was categorized into low, some, or high risk based on domain-specific point thresholds. In general, studies that scored 3–4 points in selection, 2 points in comparability, and 2–3 points in outcome assessment were considered at low risk. Studies with a total NOS score of 7 or higher were deemed to be of high methodological quality. Beyond the NOS, we performed a granular assessment of confounding domains, as emphasized by the ROBINS-I tool, to evaluate the risk of 'confounding by indication.' We systematically extracted the specific adjustment methods (e.g., Propensity Score Matching, multivariate regression) and the list of covariates controlled for in each study.

### Certainty of evidence assessment

The certainty of evidence for each outcome was evaluated using the GRADE approach, facilitated by GRADEgpt. The domains assessed included risk of bias, inconsistency, indirectness, imprecision, and publication bias. Evidence was graded as high, moderate, low, or very low. The GRADEpro tool was used to generate a Summary of Findings table, incorporating pooled effect estimates from the meta-analysis.

### Primary outcome (%TBWL over time: ESG vs IGB)

The primary outcome was the %TBWL comparing ESG and IGB. For the main analysis, we extracted the %TBWL at the longest available follow-up reported in each study to ensure consistency and avoid selective timepoint bias.

To evaluate temporal trends in treatment effect, we conducted stratified meta-analyses at predefined timepoints (1, 3, 6, and 12 months), along with a subgroup analysis comparing studies with follow-up durations ≤ 3 months versus > 3 months.

We also performed a random-effects meta-regression to assess the association between follow-up duration (in months) and the between-group difference in %TBWL. Each study contributed a single data point based on its longest available follow-up, and follow-up duration was modeled as a continuous moderator. The regression coefficient estimated the change in treatment effect per additional month of follow-up. Statistical significance was defined as a two-tailed p-value < 0.05. Subgroup analyses based on follow-up duration were considered exploratory. No adjustments for multiple comparisons were applied; therefore, these findings should be interpreted as hypothesis-generating.

### Secondary outcomes (Baseline BMI difference meta-regression; adverse events analysis)

Secondary outcomes included (1) the relationship between baseline BMI difference and treatment effect on %TBWL, and (2) the incidence of adverse events in ESG versus IGB. A random-effects meta-regression was conducted using baseline BMI difference as the covariate and %TBWL difference as the outcome.

For safety analysis, reported adverse events were extracted and categorized. A pooled odds ratio comparing ESG and IGB was calculated using a random-effects model, with heterogeneity assessed by the I^2^ statistic.

### Data extraction and management

For each study comparing ESG and IGB, data were systematically extracted using a standardized form. Extracted information included study characteristics (author, year, design), participant demographics (age, sex, baseline BMI), treatment details, follow-up duration, and outcomes such as percentage of (%TBWL and adverse events. Effect size directionality was carefully verified for consistency across studies.

For the primary analysis, when studies reported multiple post-treatment timepoints, we extracted data from the longest available follow-up to represent the overall treatment effect. In addition, stratified analyses were performed at predefined timepoints (1, 3, 6, and 12 months) to evaluate the trajectory of %TBWL over time.

To further explore factors influencing outcomes, we performed secondary analyses including a random-effects meta-regression assessing the association between baseline BMI difference and between-group %TBWL, as well as a meta-analysis of adverse events. Adverse events were extracted from each study and categorized based on reported definitions. A pooled odds ratio comparing ESG and IGB was calculated using a random-effects model, with heterogeneity assessed using the I^2^ statistic.

### Statistical analysis

Given the anticipated heterogeneity across studies, a random-effects model was applied using Comprehensive Meta-Analysis software (version 4, Biostat, Englewood, NJ, USA)^18^. For continuous outcomes, including %TBWL, mean differences with corresponding 95% confidence intervals (CIs) were calculated. For dichotomous outcomes such as adverse events, results were synthesized as odds ratios (ORs) with 95% CIs.

For the pooled analysis, data were extracted as means and standard deviations (SDs). We observed that all included studies directly reported weight loss outcomes as %TBWL with corresponding means and SDs, precluding the need for statistical estimation from medians or interquartile ranges.

In addition, random-effects meta-regression analyses were conducted to explore whether the between-group difference in baseline BMI and the duration of follow-up influenced the treatment effect on %TBWL. The assumption of a linear relationship between moderators and effect size was assessed through visual inspection of the weighted scatter plots. Given the limited number of included studies (k = 6), non-linear models were not applied to prioritize model parsimony and avoid overfitting.

Statistical heterogeneity was assessed using the I^2^ statistic and Cochran’s Q test, with I^2^ values of 25%, 50%, and 75% interpreted as low, moderate, and high heterogeneity, respectively^19^. Sensitivity analyses were conducted by sequentially removing one study at a time to evaluate the robustness of the pooled results^20^. To assess the influence of individual studies on the meta-regression outcomes, a leave-one-out sensitivity analysis was performed for each moderator (follow-up duration and baseline BMI difference) by sequentially omitting one study at a time and re-calculating the regression coefficients and p-values. Publication bias was assessed through visual inspection of funnel plots and followed Cochrane Handbook recommendations^21^.

#### Use of AI language tools

Language editing and manuscript refinement were assisted by ChatGPT (OpenAI, San Francisco, CA), a large language model-based AI tool. The authors reviewed and revised the output to ensure accuracy and appropriateness of the content. No AI-generated content was used for data analysis or interpretation.

## Results

### Study selection and characteristics

**Fig. 1:**
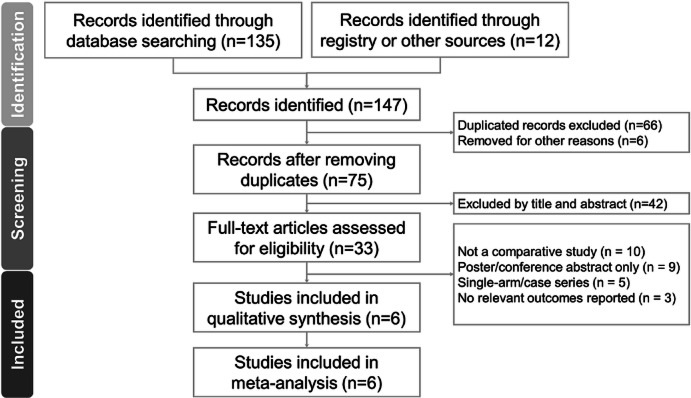
The PRISMA flowchart.

Figure 1 presents the PRISMA flow diagram summarizing the literature search and selection process. After removing duplicates and excluding irrelevant records based on titles and abstracts, six comparative cohort studies were included in the final analysis. A list of studies excluded at the full-text review stage, along with reasons for exclusion, is provided in Supplementary Table S3.

The six eligible studies encompassed a total of 5,330 participants, with a pooled mean age of 46.44 ± 11.09 years and a mean BMI of 36.42 ± 6.21 kg/m^2^. Of these, 80.2% (n = 4278) were female. Study durations ranged from 1 to 12 months, and all participants were diagnosed with obesity. Regarding the specific IGB devices utilized, the majority of included studies employed fluid-filled balloons. Limas et al. and Rapaka et al. used the Orbera system exclusively^14,16^. Kozłowska-Petriczko et al. utilized Orbera and Orbera365^13^. Fayad et al. and Lopez-Nava et al. utilized a combination of Orbera and ReShape Integrated Dual Balloon System, though Lopez-Nava et al. explicitly excluded swallowable balloons (Elipse)^11,15^. Consequently, the IGB cohort in this meta-analysis predominantly represents fluid-filled devices. A summary of the included trials is provided in Table 1.

**Table 1:** Summary of the retrieved studies comparing the effects of ESG and IGB in patients with obesity.

First author & year	Country	Population	Parti cipants (female/male)	Age ^1^	BMI (kg/m^2^)	Study design	IGB Device Type	Follow-up Timepoints	Funding/grants/support
Fayad^11^	USA	Patients with obesity treated with IGB/ESG	ESG: 34/24 IGB: 46/1	48.2 ± 11.8^2^ 47.7 ± 12.4	41.5 ± 8.2 34.5 ± 6.7	Retrospective, single-center cohort study	Orbera (Fluid-filled)	1, 3, 6 and 12 months	N/A
Gudur^12^	USA Canada	Patients with obesity treated with IGB/ESG	ESG: 1653/345 IGB: 1633/365	46.94 ± 11.14 ^2^ 46.82 ± 11.66	36.36 ± 6.19 36.17 ± 6.16	Retrospective cohort with propensity-matched analysis using the MBSAQIP	Orbera (Fluid-filled)	1 month	N/A
Petriczko (2022)	Poland	Patients with obesity treated with IGB/ESG	ESG: 29/13 IGB: 104/20	^43^4^.^2^8^.0^±^ ± ^8^9^.^.^8^9	44.8 ± 4.5 34.2 ± 5.0	Retrospective, cohort study	Orbera & Orbera365 (Fluid-filled)	6 and 12 months	N/A
Limas^14^	Indonesia	Patients with obesity (BMI > 27 for IGB, > 30 for ESG)	ESG: 17/3 IGB: 24/7	40.05 ± 10.54 ^2^ 38.67 ± 8.41	31.12 ± 5.88 31.16 ± 4.39	Retrospective cohort study	Orbera (70%) & ReShape (30%) (Both Fluid-filled)	1 week, 1 and 3 months	N/A
Lop ez-Nava^15^	Spain	Patients with obesity treated with IGB/ESG	Total: 691/271	ESG: 45.9 ± 9.6^2,3^ Orbera: 42.9 ± 11 Reshape:42.3 ± 12.8	ESG:38.3 ± 5.7 Orbera: 37.6 ± 6.7 Reshape: 38.4 ± 5.2	Prospective data collection, retrospective	Orbera (81%) & ReShape (19%) (Elipse excluded)	6 and 12 months	N/A
Rapaka^16^	Spain	Patients with obesity treated with IGB/ESG	ESG: 29/3 IGB: 18/0	47.69 ± 5.06^2^ 41.06 ± 8.81	41.21 ± 5.38 34.50 ± 4.46	Prospective cohort study	Not specified	3 months, 6 months	Medtronic, Apollo Endosurgery, USGI, Cairn Diagnostics, Aspire, Spatz Medical, NIH-NIDDK

### Risk of bias and quality of evidence

**Fig. 2:**
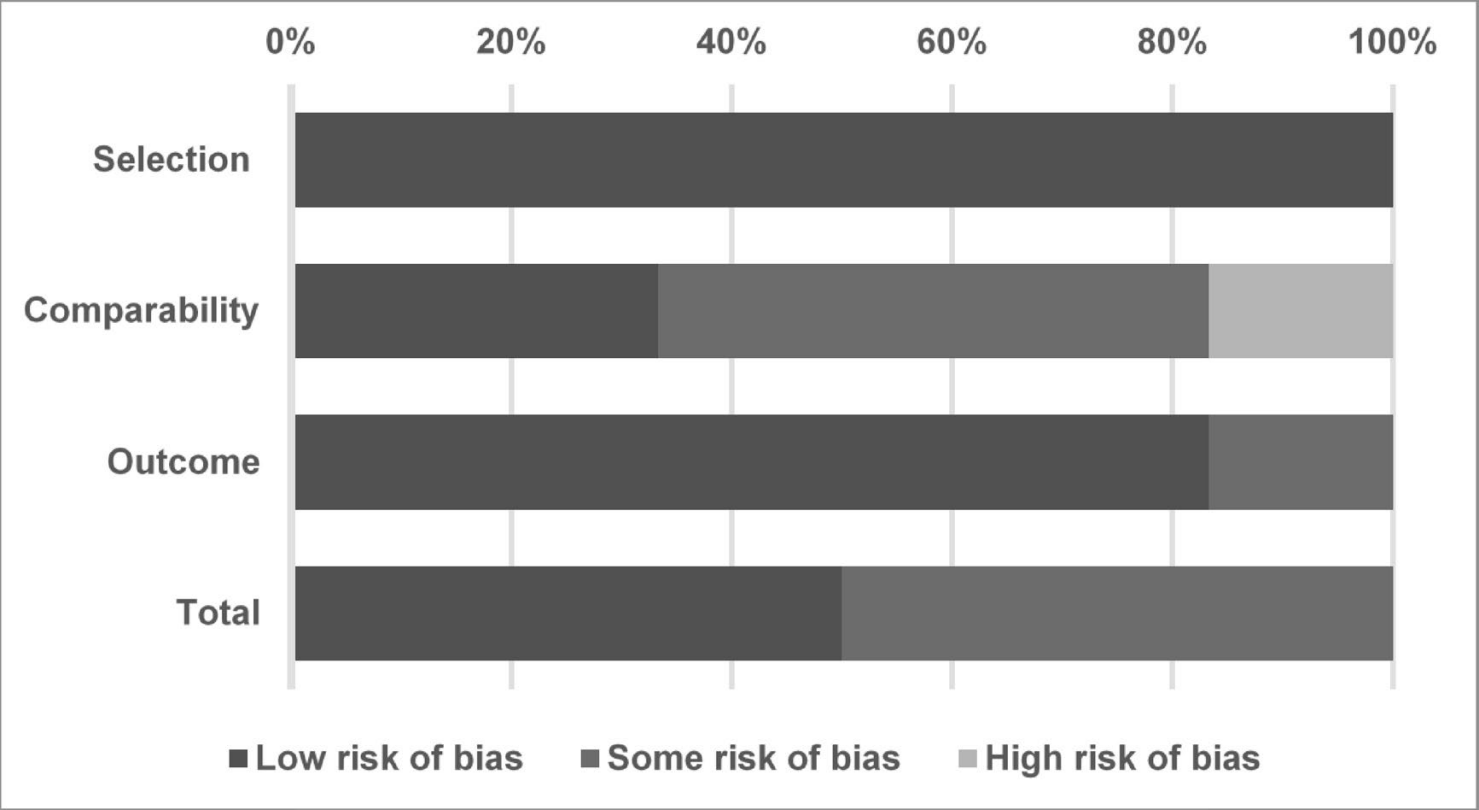
Summary of quality assessment of studies included in the meta-analysis using the NOS. For graphical presentation of risk of bias, individual NOS domains were categorized into low, some, or high risk based on domain-specific scores: Selection (maximum 4 points), Comparability (2 points), and Outcome (3 points). Domains scoring ≥ 75% of the maximum were considered low risk, 50–74% as some risk, and < 50% as high risk.

Among the six included studies, two (Gudur^12^ and Nava 2019) were rated as low risk of bias (NOS score: 8–9), with strong methodology, well-matched cohorts, and adequate follow-up^12,15^. The graphical summary of the quality assessment and the detailed scoring for each domain are presented in Fig. 2 and Table 2, respectively. Three studies (Fayad^11^, Petriczko 2022, and Limas^14^) showed moderate risk (score: 6–7), primarily due to baseline imbalances, incomplete follow-up, or limited confounder control^11,13,14^. One study (Rapaka^16^) had a moderate-to-high risk of bias (score: 5), with minimal adjustment for baseline differences and potential selection bias^16^. Common limitations included retrospective design, short or heterogeneous follow-up durations, and varying loss-to-follow-up rates, which may impact internal validity and generalizability.

**Table 2:** Detailed quality assessment of included studies using Newcastle–Ottawa Scale tool.

First Author Year	Study design	Selection	Comparability	Outcome	Total	Subjective evaluation
Fayad^11^	Retrospective	4	1	2	7	Low risk of bias
Gudur^12^	Retrospective	4	2	3	9	Low risk of bias
Petriczko (2022)	Retrospective	3	1	2	6	Moderate risk of bias
Limas^14^	Retrospective	4	1	1	6	Moderate risk of bias
Lopez-Nava^12^	Retrospective	4	2	2	8	Low risk of bias
Rapaka (2022)	Prospective	3	0	2	5	Moderate risk of bias

To enhance transparency regarding the risk of "confounding by indication"—a critical domain in the ROBINS-I framework—we further assessed the specific adjustment strategies used in each study. As detailed in Supplement Table S5, Gudur et al.^12^ minimized confounding by indication through 1:1 propensity score matching (PSM) for age, sex, race, baseline BMI, and multiple comorbidities. Fayad et al. and Lopez-Nava et al. utilized multivariate regression to adjust for critical covariates including baseline BMI. For studies without formal statistical adjustment, such as Limas et al. and Kozłowska-Petriczko et al., the risk of “at-intervention” confounding was mitigated through the implementation of identical, standardized multidisciplinary nutritional and behavioral protocols for both ESG and IGB cohorts.

### Primary outcome (%TBWL over time: ESG vs IGB)

**Fig. 3:**
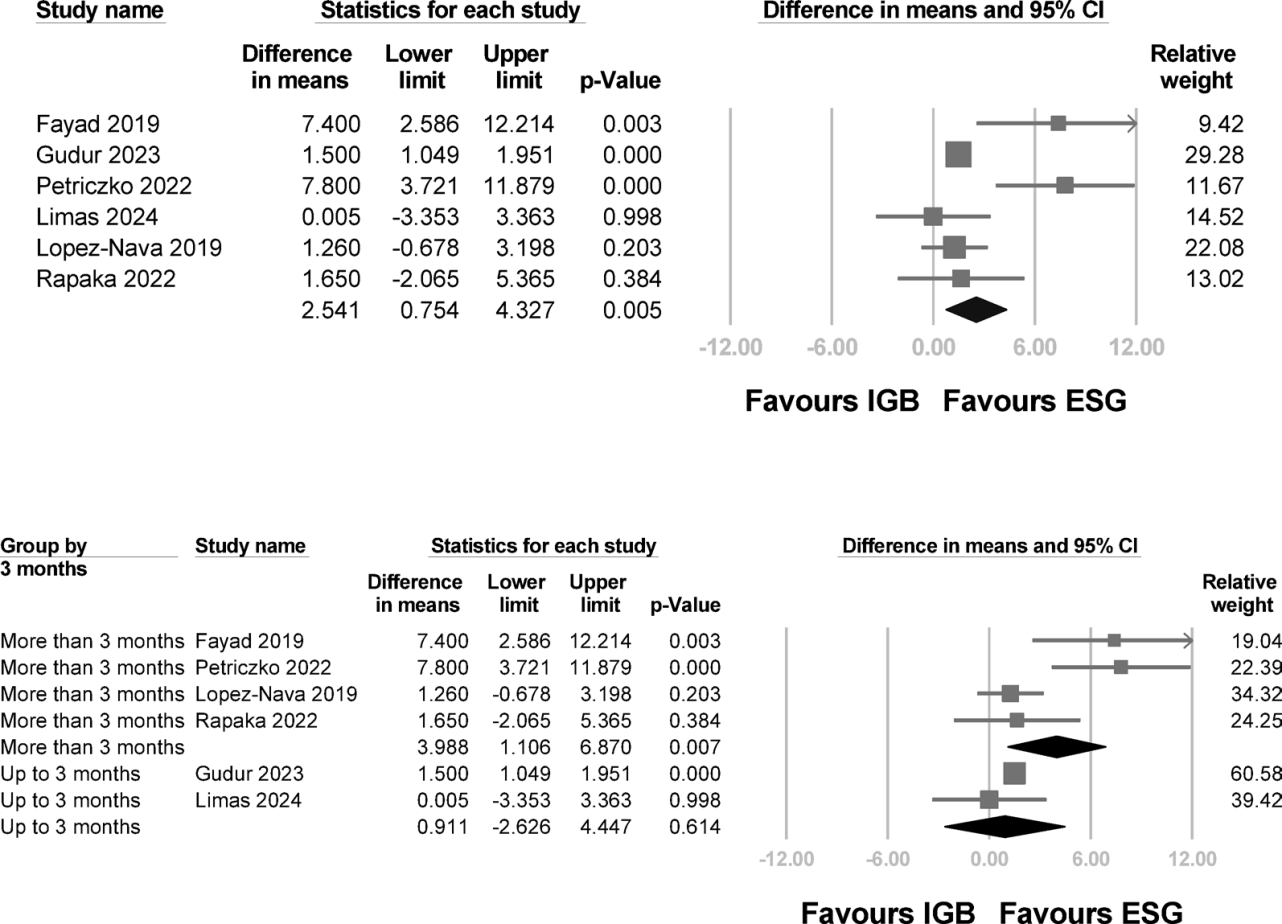
Forest plots of the percentage of %TBWL at the longest available follow-up across studies comparing ESG and intragastric balloon IGB. (A) Pooled analysis showing a significantly greater %TBWL with ESG compared to IGB (mean difference: 2.541; 95% CI 0.754 to 4.327; p = 0.005), with moderate-to-high heterogeneity (I^2^ = 67.98%). (B) Subgroup analysis stratified by follow-up duration (≤ 3 months vs. > 3 months). The pooled effect in studies with > 3 months of follow-up showed a larger mean difference (3.988; 95% CI 1.106 to 6.870; p = 0.007), favoring ESG, while the effect in studies with ≤ 3 months follow-up was not statistically significant.

Pooled analysis of six studies, using the longest available follow-up from each, showed that ESG was associated with significantly greater %TBWL compared to IGB (mean difference: 2.541; 95% CI 0.754 to 4.327; p = 0.005), with moderate-to-high heterogeneity (I^2^ = 67.98%) (Fig. 3A). Leave-one-out sensitivity analysis confirmed the robustness of the result; as shown in Supplementary Figure S1, the effect size remained significant and consistently favored ESG regardless of which study was excluded.

To explore the temporal dynamics of efficacy, subgroup analysis stratified by follow-up duration demonstrated a significant benefit of ESG in studies with follow-up longer than 3 months (mean difference: 3.988; 95% CI 1.106 to 6.870; p = 0.007), whereas no significant difference was observed in studies with 3 months or shorter follow-up (mean difference: 0.911; 95% CI –2.626 to 4.447; p = 0.614) (Fig. 3B).

**Fig. 4:**
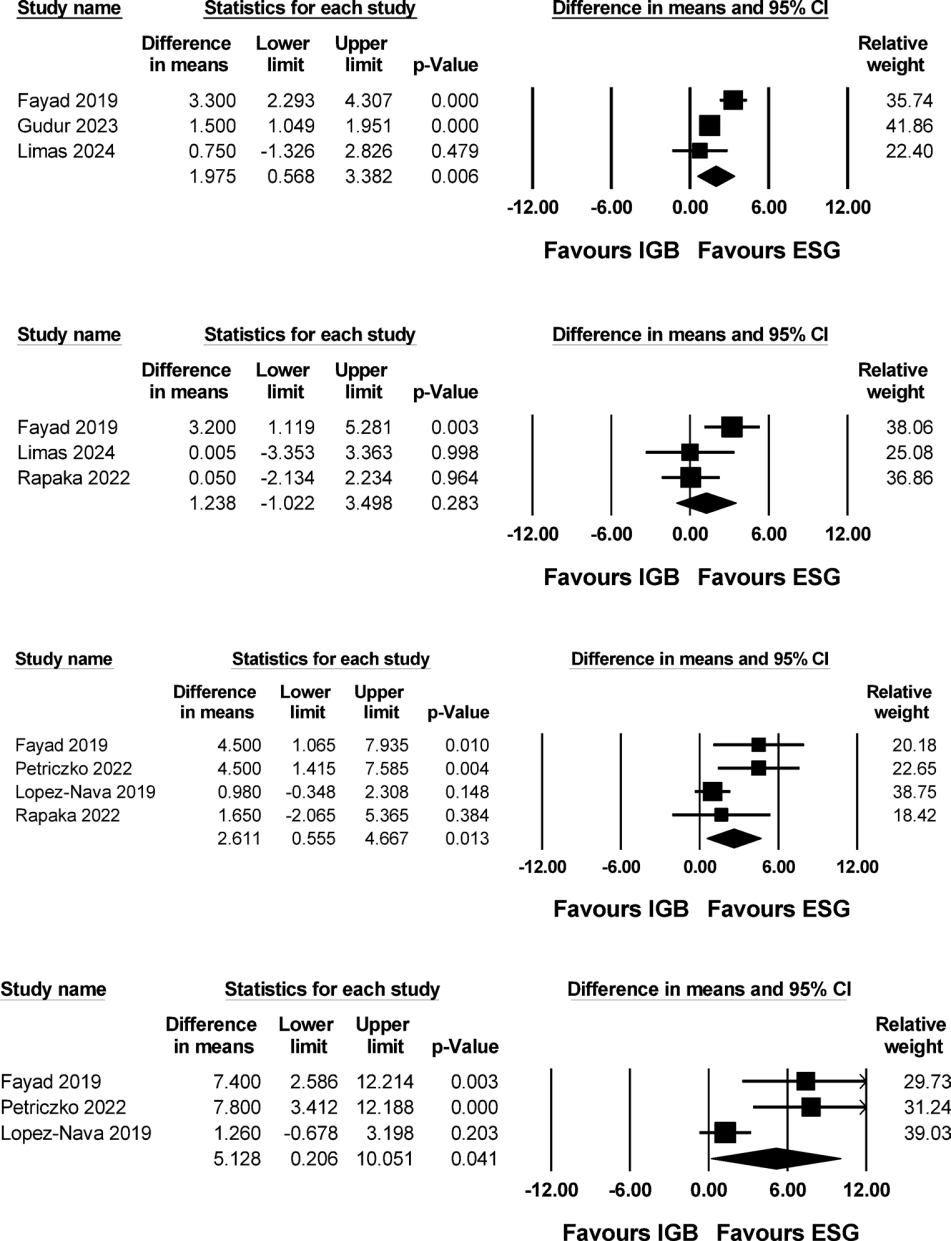
Forest plots of the percentage of total body weight loss (%TBWL) at 1, 3, 6, and 12 months comparing ESG versus IGB. (A) 1 month, pooled mean difference: 1.975 (95% CI 0.568–3.382), heterogeneity I^2^ = 82.1% (B) 3 months, pooled mean difference: 1.238 (95% CI –1.022 to 3.498), heterogeneity I^2^ = 60.0% (C) 6 months, pooled mean difference: 2.611 (95% CI 0.555–4.667), heterogeneity I^2^ = 55.8% (D) 12 months, pooled mean difference: 5.128 (95% CI 0.206–10.051), heterogeneity I^2^ = 81.6%

Further stratification by specific timepoints showed a consistent trend favoring ESG. At 1 month, the pooled mean difference was 1.975 (95% CI 0.568 to 3.382; I^2^ = 82.1%). At 3 months, the difference was not statistically significant (1.238; 95% CI –1.022 to 3.498; I^2^ = 60.0%). By 6 months, ESG demonstrated a significant advantage (2.611; 95% CI 0.555 to 4.667; I^2^ = 55.8%), which became more pronounced at 12 months (5.128; 95% CI 0.206 to 10.051; I^2^ = 81.6%) (Fig. 4).

**Fig. 5:**
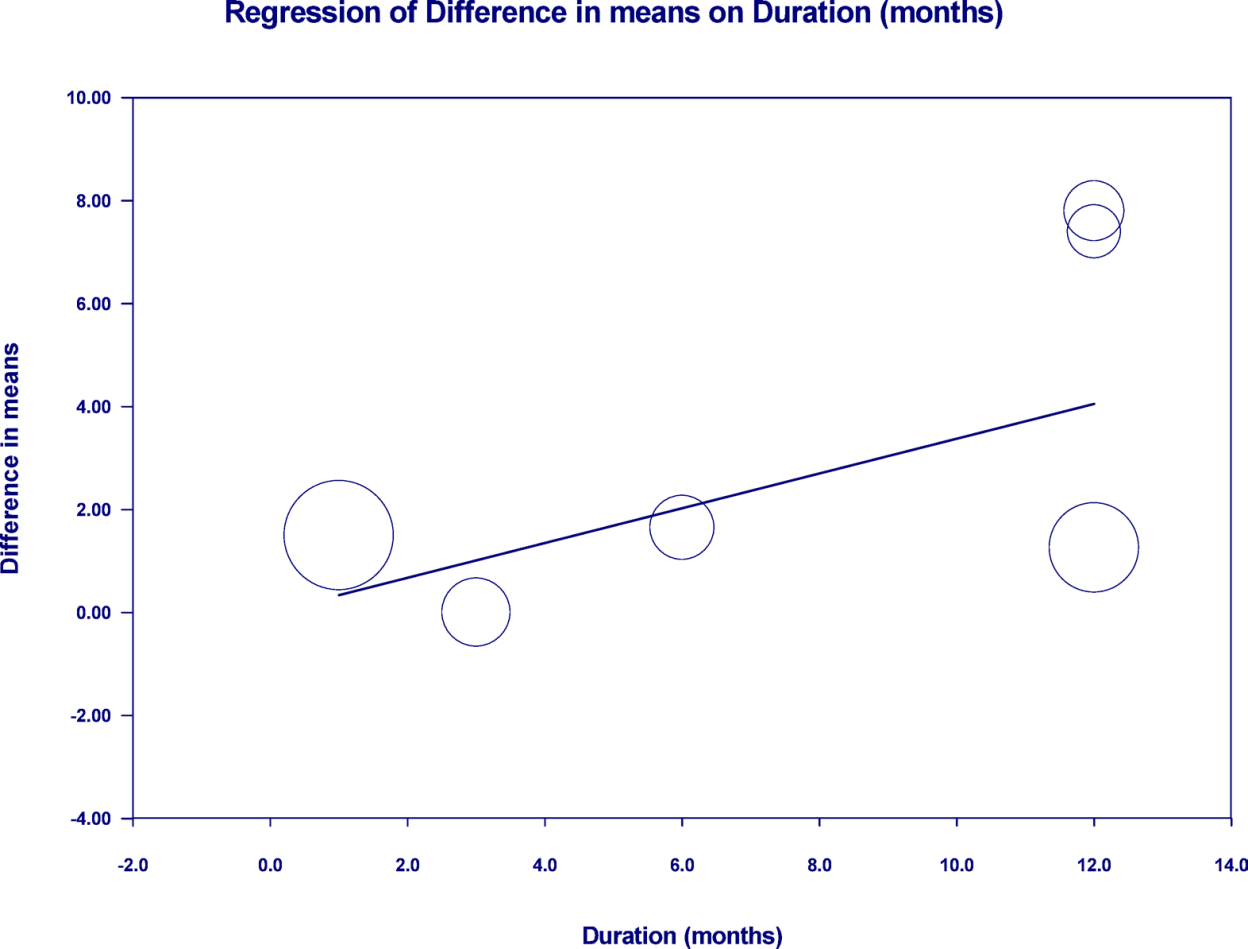
Meta-regression analysis evaluating the association between follow-up duration (months) and the difference in %TBWL between ESG and intragastric balloon IGB. A significant positive relationship was observed (coefficient = 0.3377, p = 0.0006), indicating that longer follow-up duration is associated with a greater treatment effect favoring ESG.

A random-effects meta-regression further confirmed a significant positive association between follow-up duration and %TBWL difference (coefficient = 0.3377, *p* = 0.0006), indicating that the benefit of ESG over IGB increases with longer follow-up (Fig. 5).

Assessment of publication bias was performed through visual inspection of the funnel plot (Supplementary Figure S2), which revealed slight asymmetry. Although Egger’s regression test was conducted, the result (*p* = 0.2916) should be interpreted with caution due to limited statistical power given the small number of included studies (n = 6). Therefore, the possibility of publication bias or small-study effects cannot be excluded.

### Secondary outcomes (Baseline BMI difference meta-regression; Adverse events analysis)

**Fig. 6:**
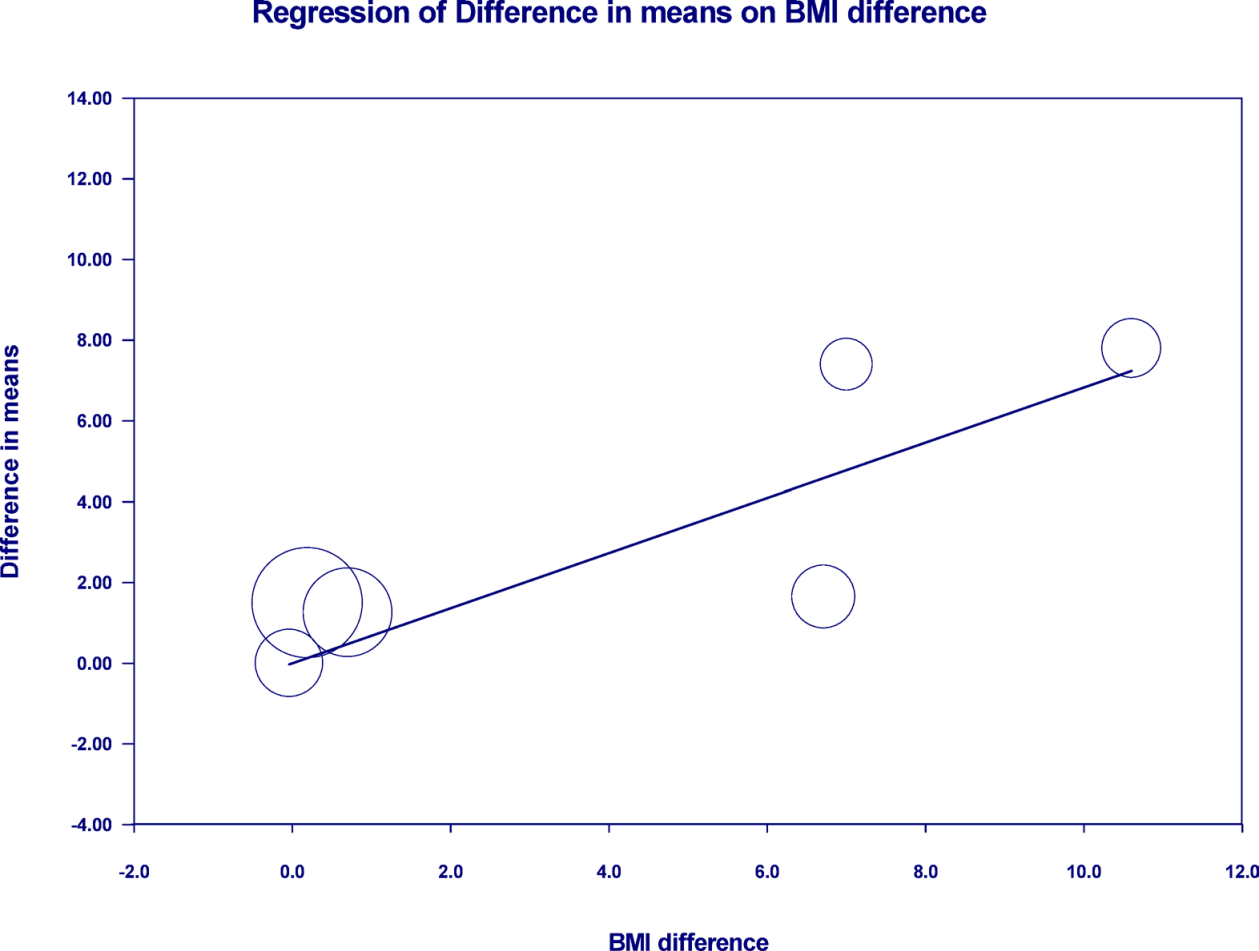
Meta-regression analysis assessing the association between baseline BMI difference (ESG minus IGB) and the between-group difference in total body weight loss (%TBWL). A significant positive correlation was observed (coefficient = 0.6827, *p* = 0.0001), indicating that for every 1 kg/m^2^ higher baseline BMI in the ESG group compared to the IGB group, the.

To further explore factors influencing treatment efficacy and safety, we conducted a meta-regression and adverse events analysis. Meta-regression showed a significant positive association between baseline BMI difference and %TBWL (coefficient = 0.6827, *p* = 0.0001), indicating that ESG provides greater weight loss benefit in patients with higher baseline BMI (Fig. 6). Leave-one-out sensitivity analysis confirmed the robustness of these findings, as the positive associations for both moderators remained statistically significant (*p* < 0.05) regardless of which study was excluded from the models.

Regarding safety, four studies reported adverse events, showing comparable overall safety profiles between the two modalities. In Fayad et al. (2019), adverse events occurred in 17% of the IGB group compared to 5.2% in the ESG group (*p* = 0.048), with IGB events primarily involving nausea, vomiting, and device-related issues leading to early removal. Conversely, Gudur et al.^12^ observed a slightly higher rate of serious adverse events in the ESG group (0.9% vs. 0.3% for IGB), though this difference was not statistically significant. While Petriczko et al. (2022) and Limas et al.^14^ reported no adverse events with ESG, IGB intolerance necessitated early removal in 10.3% and 16.1% of patients, respectively.The pooled odds ratio (Supplementary Figure S3) revealed no statistically significant difference in adverse event rates between the two procedures (OR = 0.422; 95% CI 0.062–2.884; *p* = 0.379). These results should be interpreted with caution due to the heterogeneous definitions of adverse events and inconsistent reporting across studies. Overall, while ESG provides superior weight loss efficacy in patients with higher BMI, it demonstrates a comparable safety profile to IGB, with observed trends suggesting potentially better patient tolerability regarding device-related issues.

Furthermore, to evaluate the robustness of this association and mitigate potential confounding from baseline imbalances, sensitivity meta-regressions were conducted. When using the mean baseline BMI of the ESG cohort as the moderator, the positive association remained significant (*p* = 0.0018; Supplementary Figure S4). A similar significant trend was observed using the overall mean BMI (*p* = 0.0031; Supplementary Figure S5). These analyses support the study-level finding that ESG’s therapeutic advantage is more pronounced in cohorts with more severe obesity.

### Certainty of evidence

Based on the GRADE assessment, the certainty of evidence for the primary outcome (%TBWL at longest follow-up) was rated as moderate, downgraded for inconsistency due to moderate-to-high heterogeneity (I^2^ = 67.98%). Evidence for time-dependent treatment effects and baseline BMI influence was graded as **low**, primarily due to observational study designs and risk of confounding. (Supplementary Table S4).

## Discussion

### Principal findings

This meta-analysis of six comparative cohort studies comparing ESG and IGB revealed several key findings. First, ESG was associated with significantly greater %TBWL than IGB, based on the longest available follow-up in each study. This advantage remained robust across sensitivity analyses and became more pronounced over time. Stratified analysis by specific timepoints suggests that 3 months may represent a clinical inflection point: the difference in %TBWL between ESG and IGB was not statistically significant at 3 months, and most individual studies at this timepoint showed overlapping confidence intervals. However, by 6 and 12 months, ESG demonstrated a significantly greater effect, suggesting a time-dependent divergence in efficacy.

Second, meta-regression analyses showed that the benefit of ESG over IGB was positively associated with both follow-up duration and baseline BMI difference. This implies that ESG provides greater relative weight loss in individuals with higher initial BMI and that its effectiveness becomes more apparent with extended follow-up.

Third, although the pooled analysis of adverse events did not reach statistical significance (*p* = 0.379) the overall safety profile of ESG was found to be comparable to that of IGB. In individual cohorts, ESG was associated with fewer intolerance-related events and lower rates of early device removal. For instance, two studies reported no adverse events in the ESG arm, whereas IGB groups experienced adverse effects—primarily device intolerance—that necessitated medical intervention or early removal^13,14^. Together, these findings support the superior efficacy of ESG over IGB, particularly in patients with higher BMI and beyond the 3-month threshold, while maintaining a comparable and acceptable safety profile.

### Mechanisms

Several mechanisms may underlie the superior and progressively increasing weight loss observed with ESG compared to IGB. Unlike IGB, which exerts a temporary space-occupying effect and is removed after approximately six months, ESG creates a durable anatomical modification by reducing gastric volume and restricting gastric accommodation through full-thickness suturing along the greater curvature^22,23^ . While both procedures induce early satiety, ESG’s structural changes also lead to mild but sustained delays in gastric emptying, whereas IGB produces more pronounced, yet transient, delays that reverse after device removal^16,24^.

In the short term, IGB may result in greater initial weight loss due to stronger gastric emptying delays, but its effects on gut hormones are inconsistent and short-lived. Studies have reported variable changes in ghrelin and transient decreases in leptin, with limited data available on GLP-1 and PYY^25,26^. In contrast, ESG was initially thought to have limited hormonal impact during early follow-up; however, recent prospective evidence has demonstrated significant increases in GLP-1, PYY, and fasting ghrelin levels at 18 months post-procedure, suggesting progressive neurohormonal adaptations that support long-term metabolic benefits^24^. These hormonal shifts, alongside persistent reductions in leptin, may enhance satiety, regulate energy balance, and contribute to ESG’s durable efficacy^7^.

Furthermore, ESG induces both delayed gastric emptying and sustained reduction in gastric accommodation. While the delay in gastric emptying has been associated with short-term weight loss, this effect is significantly less pronounced than that seen with IGB and contributes less to early weight reduction^16,24^. In contrast, the persistent reduction in gastric capacity after ESG, along with progressive hormonal adaptations, appears to play a dominant role in long-term weight control. On the other hand, IGB primarily relies on pronounced but temporary delays in gastric emptying, and its effects often diminish after balloon removal, frequently leading to weight plateau or regain^27^.

These differences in anatomical persistence, physiological adaptation, and endocrine response likely explain the divergence in %TBWL between ESG and IGB observed in our meta-analysis, becoming especially apparent beyond the 3-month mark. ESG’s evolving gut–brain axis effects and structural integrity offer a more sustained therapeutic benefit compared to the short-term mechanisms of IGB^5^.

### Implications for clinical practice and research

The findings of this meta-analysis have important implications for both clinical decision-making. Specifically, baseline BMI appears to be a critical determinant for procedure selection. Our analysis suggests that ESG should be preferentially recommended for patients with Class III obesity (BMI ≥ 40 kg/m^2^). Studies involving ESG cohorts with mean BMIs exceeding 40 kg/m^2^ consistently demonstrated the superior efficacy of ESG^11–13^. Conversely, Limas et al. reported comparable outcomes between ESG and IGB in a population with Class I obesity (mean BMI ~ 31 kg/m^2^), suggesting that IGB remains a competitive option for patients with lower BMI ranges (30–35 kg/m^2^)^14^.

Therefore, a stratified approach is advisable: For patients with BMI 30–40 kg/m^2^, IGB may be considered a first-line intervention, offering effective weight loss with lower procedural complexity and cost. However, for patients with BMI ≥ 40 kg/m^2^, ESG represents the more robust option, offering significantly greater %TBWL and superior durability needed to address severe obesity. The time-dependent superiority of ESG observed in this analysis highlights the importance of individualized treatment planning based on patient characteristics and weight loss goals. Given the progressive enhancement of %TBWL beyond 3 months and the favorable safety profile of ESG, it may be preferable over IGB in patients who are candidates for non-surgical bariatric therapies and who prioritize sustained weight reduction. However, anatomical durability alone does not guarantee long-term success. The management of patients post-ESG requires a paradigm shift from acute intervention to chronic disease management. Evidence from included studies indicates that weight loss outcomes are strongly predicted by adherence to multidisciplinary team (MDT) follow-up rather than the procedure alone^11,15^. Lopez-Nava et al. found that high attendance at nutritional and psychological counseling sessions was an independent predictor of achieving > 10% TBWL at 1 year^15^. Similarly, Limas et al. highlighted that frequent MDT interaction facilitates the early identification of patients at risk of weight recidivism^14^. Consequently, ESG should be implemented within a comprehensive program that provides ongoing nutritional guidance and psychological support to reinforce behavioral changes, ensuring that the physiological advantage of the procedure translates into sustained clinical benefit. Consistent with these findings, a recent review emphasizes that the future of endoscopic bariatric therapies lies in strengthened interdisciplinary collaboration—integrating medicine, nutrition, and psychology—to develop comprehensive strategies that maximize therapeutic benefits. Consequently, ESG should be implemented within such comprehensive programs, potentially synergistic with pharmacotherapy, to ensure that the physiological advantage of the procedure translates into sustained clinical benefit^28^.

While a formal cost-effectiveness analysis was beyond the scope of this study, the economic implications of ESG versus Intragastric Balloon IGB warrant discussion. Generally, ESG incurs a higher upfront cost due to the requirement for general anesthesia, specialized suturing devices, and longer operative times compared to IGB. For instance, previous reports have noted a program cost of approximately $16,000 for ESG versus $8,000 for IGB, while others observed a similar disparity ($10,150 vs. $3,650)^11,14^.

However, the relative value of IGB is often attenuated by downstream costs. Unlike ESG, IGB requires a mandatory second endoscopic procedure for removal, incurring additional anesthesia and facility fees. Furthermore, data from the included studies suggest that IGB patients may experience higher rates of outpatient treatments for dehydration and re-interventions within 30 days, often driven by device intolerance. Notably, the rate of early device removal (up to 3.7% in some cohorts) represents a significant cost burden for a truncated therapeutic benefit^12^. Finally, cost-effectiveness is intrinsically linked to durability. As weight recidivism is more common after IGB removal, whereas ESG offers a durable anatomical modification, ESG may offer superior long-term value by potentially obviating the need for repeat interventions or future conversion to surgery.

From a research perspective, our findings underscore the need for longer-term comparative studies to further validate the durability of ESG’s effects beyond one year and to better characterize its metabolic benefits, including endocrine adaptations. Future trials should also standardize outcome reporting at fixed intervals (e.g., 6, 12, and 24 months) to facilitate cross-study comparisons and meta-analytic synthesis. In addition, more granular data on patient-reported outcomes, quality of life, and cost-effectiveness are needed to support evidence-based guidelines. Investigations into hormonal profiles and gastric physiology following ESG may also uncover mechanistic biomarkers predictive of treatment response, helping to optimize patient selection and personalize care. While this meta-analysis confirms the short-term superiority of ESG, significant knowledge gaps regarding long-term efficacy and safety remain. The current evidence base is limited by a lack of comparative data beyond one year, making conclusions regarding the multi-year sustainability of ESG somewhat preliminary. First, the durability of the physiological mechanisms identified by Rapaka et al. requires longitudinal assessment. Specifically, it is unknown if the reduction in maximum tolerated volume (MTV) achieved by ESG persists beyond 1–2 years or if physiological adaptation (e.g., gastric remodeling or suture loosening) eventually diminishes the satiation effect^16^.

Second, the management of weight recidivism differs fundamentally between the two modalities. Fayad et al. and Kozłowska-Petriczko et al. emphasize that weight regain is a common sequela of IGB removal, necessitating a pre-planned maintenance strategy^11,13^. In contrast, for ESG, the long-term safety of revisional interventions remains an open question. As noted by Fayad et al., there is a theoretical concern that ESG may induce perigastric scarring that could complicate future conversion to laparoscopic sleeve gastrectomy or gastric bypass, whereas IGB preserves surgical planes^11^. Future studies must address these ‘sequencing’ questions—including the potential synergy of combining ESG with pharmacotherapy as highlighted by the IFSO 2024 Position Statement—to optimize long-term chronic obesity management^5^.

Our findings align closely with and support current international recommendations, specifically the 2022 American Society for Metabolic and Bariatric Surgery (ASMBS) and International Federation for the Surgery of Obesity and Metabolic Disorders (IFSO) Indications for Metabolic and Bariatric Surgery^29^. These updated guidelines lowered the threshold for metabolic intervention, recommending it for individuals with BMI ≥ 35 kg/m^2^ regardless of comorbidities and considering it for those with BMI 30–34.9 kg/m^2^ who do not achieve substantial weight loss with non-surgical methods^5^.

Our meta-analysis provides the evidentiary support needed to implement these guidelines using endoscopic therapies. Specifically, our finding that ESG demonstrates superior efficacy in patients with higher baseline BMI reinforces the 2024 IFSO Bariatric Endoscopy Committee Position Statement, which explicitly endorses ESG not only for Class I and II obesity but also for patients with Class III obesity who decline or are ineligible for surgical bariatric procedures^5^. While the ASMBS/IFSO guidelines highlight the substantial ‘treatment gap’ where < 1% of eligible patients undergo surgery^29^, our data suggests that ESG can effectively bridge this gap. By offering a safety profile comparable to IGB but with superior durability and weight loss magnitude—especially in the Class III obesity population highlighted by the guidelines—ESG represents a critical tool for expanding access to care consistent with the ASMBS and IFSO quality standards.

### Strengths and limitations

This meta-analysis provides an updated comparison of ESG and IGB based on comparative cohort studies. Strengths include a systematic literature search, inclusion of both efficacy and safety outcomes, and the use of stratified timepoint analyses and meta-regression to examine how follow-up duration and baseline BMI influence treatment effect—offering valuable insights for individualized care, particularly in optimizing treatment strategies for patients with higher BMI or those seeking sustained long-term weight loss. Study quality was assessed using the NOS and ROBINS-I, and additional analyses confirmed the robustness of the findings.

Limitations include the observational design of all included studies, which may introduce confounding. A primary limitation of this meta-analysis is the reliance on comparative cohort studies, as no direct head-to-head RCTs met inclusion criteria. This design inherently limits causal inference and increases susceptibility to selection bias and confounding by indication. However, several included studies employed rigorous statistical techniques to mitigate these biases. Gudur et al. utilized propensity score matching (1:1) to balance baseline covariates, while Fayad et al. and Lopez-Nava et al. used multivariate regression to adjust for potential confounders^11,12,15^. Furthermore, our findings derived from real-world data display remarkable concordance with the recent network meta-analysis of RCTs by Mrad et al.^30^, which also identified ESG as superior to fluid-filled balloons regarding weight loss efficacy^30^. This alignment suggests that the observational data reported here likely reflects true therapeutic differences rather than unmeasured confounding. Nevertheless, results should be interpreted as associations within a real-world clinical context.

Although unmeasured confounders such as lifestyle adherence could influence outcomes, several included studies (Fayad et al., Kozłowska-Petriczko et al., Lopez-Nava et al.) mitigated this by utilizing identical post-procedure nutritional and behavioral protocols for both groups^11,13,15^. Similarly, to minimize operator-dependent bias, procedures in these single-center studies were typically performed by the same endoscopists^11,15^. Regarding generalizability, we acknowledge that several included studies were conducted in specialized, high-volume centers with significant endoscopic expertise (e.g., Fayad et al. and Lopez-Nava et al.), which may reflect "best-case" clinical outcomes. However, this limitation is partially offset by the inclusion of the MBSAQIP registry analysis by Gudur et al., which aggregated data from over 800 centers across North America. This large-scale dataset suggests that the safety and efficacy trends observed in specialized centers are reproducible in broader clinical practice. Nevertheless, as noted by the IFSO Bariatric Endoscopy Committee, ESG remains a technically demanding procedure, and outcomes in lower-volume community settings may vary depending on the local learning curve and adherence to standardized protocols. However, selection bias cannot be fully excluded. A significant limitation of this analysis is the variability in follow-up duration and inconsistent reporting of timepoints across included studies. For instance, the largest included study by Gudur et al. provided data only up to 30 days, whereas long-term data (≥ 12 months) were derived from a smaller subset of studies (e.g., Fayad et al., Lopez-Nava et al.)^11,15^. This introduces a risk that early timepoint estimates are driven by registry data while later timepoints reflect single-center experiences. We attempted to mitigate this via stratified analyses at fixed intervals (1, 3, 6, and 12 months) to ensure comparability at each specific stage. Furthermore, the use of meta-regression allowed us to model follow-up duration as a continuous moderator, confirming that the treatment effect size is positively associated with time (*p* = 0.0006). Furthermore, we acknowledge the inherent risk of attrition bias in obesity trials, where non-responders are more likely to drop out. However, evidence from the included studies suggests that this bias may not disproportionately favor ESG. Lopez-Nava et al. reported that ESG was an independent predictor of higher follow-up adherence compared to IGB, while Fayad et al. observed that loss to follow-up in their ESG cohort often involved patients who were satisfied with their weight loss—a factor that might actually lead to an underestimation of the procedure’s true efficacy^11,15^.

Furthermore, our primary analysis relied solely on %TBWL. While we acknowledge that %EWL, comorbidity remission, and quality of life are critical patient-centered outcomes, these were not consistently evaluated or synthesized due to heterogenous reporting across included studies. For instance, the largest study by Gudur et al. was limited to 30-day outcomes, and Limas et al. reported follow-up only up to 3 months, precluding the assessment of long-term metabolic remission or durability for a large portion of the cohort^12,14^. Consequently, while %TBWL is a validated surrogate for metabolic improvement—with 5–10% loss typically sufficient to improve cardiovascular risk factors—future trials with standardized reporting of metabolic and functional endpoints are necessary to fully characterize the clinical superiority of ESG.

Nevertheless, the reduced number of studies available for the 12-month analysis may limit the precision of long-term effect estimates compared to short-term results. As noted by Fayad et al. and Limas et al., the significantly higher cost of ESG compared to IGB may select for patients with higher financial motivation^11,14^. Furthermore, regarding baseline health, studies like Gudur et al. indicated that ESG patients often had a higher burden of comorbidities (e.g., sleep apnea), and consistently higher baseline BMI^12^. While this suggests ESG treated a metabolically more complex population, Fayad et al. posit that this baseline severity might actually mask the true extent of ESG’s efficacy, as higher baseline BMI can be associated with greater difficulty in achieving high percentage weight loss^11^. Finally, while ‘IGB’ encompasses various devices, the studies included in this analysis predominantly utilized fluid-filled balloons (specifically Orbera and ReShape). Furthermore, subtle variations in procedural protocols, such as initial fill volumes and the exact timing of balloon removal—ranging from the standard 6 months to 12 months for systems like Orbera365—may further contribute to the observed heterogeneity. Studies utilizing swallowable (e.g., Elipse) or adjustable (e.g., Spatz3) balloons were either not found or excluded (e.g., Lopez-Nava et al.). Therefore, a subgroup analysis by device type was not feasible due to the lack of stratified data in primary studies, and our findings should be interpreted specifically in the context of fluid-filled balloons and may not be generalizable to other IGB types. Safety comparisons were constrained by heterogeneous adverse event definitions. While Gudur et al. reported low event rates based on strict life-threatening criteria, studies accounting for device intolerance (Fayad et al., Kozłowska-Petriczko et al.) observed disproportionately higher adverse event rates for IGB (10–17%) due to early removal. Consequently, while ESG demonstrates superior tolerability and retention, these trends require cautious interpretation due to inconsistent reporting. Heterogeneity in populations, follow-up durations, and outcome reporting could affect consistency. Furthermore, while ESG is generally standardized using the Apollo OverStitch device across the included clinical trials, minor technical variations existed. For instance, the number of sutures ranged from 4–7 in Kozłowska-Petriczko et al. to 6–10 in Rapaka et al. and Fayad et al., likely reflecting anatomical variations rather than distinct procedural methodologies^11,13,16^. However, technical granularity was unavailable for the large registry-based study (Gudur et al.), representing a potential source of unmeasured heterogeneity regarding suture patterns or provider experience^12^. A primary limitation of this meta-analysis is the small number of included studies (k = 6), which constrains statistical power and warrants cautious interpretation of secondary analyses. Specifically, the subgroup analyses stratified by follow-up duration were exploratory and conducted without adjustment for multiple testing, increasing the potential for Type I error. Similarly, the small sample size poses a risk of overfitting in the meta-regression models. Furthermore, the meta-regression regarding baseline BMI difference must be interpreted as an exploratory, study-level association rather than individual-level effect modification. Because this analysis utilized study-level means, it is subject to the ecological fallacy and may also reflect treatment selection bias—where clinicians preferentially assigned ESG to patients with higher BMI—rather than a true biological interaction. However, we proceeded with these analyses to investigate the high heterogeneity observed (I^2^ > 60). To mitigate the risk of instability, we performed leave-one-out sensitivity analyses, which confirmed that the significant associations between treatment effect and both follow-up duration (*p* = 0.0006) and baseline BMI (*p* = 0.0001) were robust and not driven by any single study. Consequently, these findings regarding time-dependent divergence and BMI influence should be interpreted as hypothesis-generating signals rather than definitive clinical conclusions, underscoring the need for validation in future large-scale trials with pre-specified long-term endpoints. Not all studies reported adverse events, and those that did used varying definitions. Follow-up was generally limited to 12 months, restricting long-term interpretation. Finally, the potential for publication bias remains an inherent limitation. Beyond the small number of studies (k = 6), our inclusion was restricted to peer-reviewed publications to ensure methodological rigor. However, this may have excluded relevant negative findings often found in grey literature or unpublished data, potentially leading to an overestimation of ESG’s efficacy. While our search strategy was comprehensive, the results should be interpreted with the understanding that 'positive-results’ bias is prevalent in the field of endobariatrics.

### Relation to prior work

Our study significantly extends the findings of previous meta-analyses. While Diab et al. demonstrated the general superiority of ESG over IGB at 1 and 6 months, their analysis lacked granular, stratified timepoint comparisons and did not examine how treatment effects evolve over time^8,9^. In contrast, we identified a clear time-dependent therapeutic advantage for ESG. Our stratified analyses at 1, 3, 6, and 12 months reveal that the 3-month mark serves as a critical clinical inflection point; the difference in %TBWL was not statistically significant prior to 3 months but became consistently and increasingly favorable to ESG thereafter.

This temporal trend is further substantiated by our meta-regression, which confirmed that longer follow-up duration is significantly associated with a greater treatment benefit for ESG. Furthermore, our results provide real-world validation for the recent network meta-analysis of randomized controlled trials^30^. While Mrad et al. utilized indirect comparisons to rank ESG as likely superior to fluid-filled balloons, our study confirms this superiority using direct head-to-head data from over 5000 patients, thereby strengthening the external validity of ESG in clinical practice.

Finally, consistent with the recent review, our data supports the positioning of ESG as a primary endobariatric therapy, particularly for patients with higher BMI who require durable weight loss^28^. Future management strategies should likely integrate these procedures into a comprehensive multidisciplinary care model to maximize long-term outcomes.

## Conclusions

Based on our synthesis of comparative cohort data, a study-level trend was observed where ESG yielded greater weight loss than IGB, especially after 3 months. Our meta-regression models further indicate that this relative advantage is positively correlated with follow-up duration and initial obesity severity of the study populations. While both interventions demonstrate comparable overall safety, ESG showed an observed trend toward better patient tolerability with fewer device-related complications and lower rates of early removal. However, these safety findings should be interpreted with caution due to the limited and inconsistent reporting of adverse events across the current literature.

These findings suggest that ESG may be the preferred non-surgical bariatric therapy for patients seeking sustained weight reduction, especially those with more severe obesity. Future research should focus on long-term outcomes beyond 12 months, the metabolic mechanisms underlying ESG’s durability, and the development of predictive models to support personalized endoscopic obesity management.

## Supplementary Information

Below is the link to the electronic supplementary material.

Supplementary Material 1

## Data Availability

The authors confirm that the data supporting the findings of this study are available within the article.
