# Supplementary material for: Meta regression of endoscopic sleeve gastroplasty versus intragastric balloon investigating influence of duration and baseline body mass index
Source: Sci Rep. 2026 Feb 4;16:7280. doi: 10.1038/s41598-026-38374-1 (PMC12923554; doi:10.1038/s41598-026-38374-1)
Supplement: Supplementary file 1 — Supplementary Material 1 [file 41598_2026_38374_MOESM1_ESM.docx]

**Supplement figure S1.** Leave-one-out sensitivity analysis for %TBWL. Forest plot showing the effect of removing each individual study on the pooled estimate of %TBWL difference between ESG and IGB. The overall treatment effect remained statistically significant in all scenarios, with mean differences consistently favoring ESG. These findings suggest that no single study disproportionately influenced the overall results, supporting the robustness of the primary analysis.

**Supplement figure S2.** Funnel plot for the 6 included trials assessing publication bias. Visual inspection reveals slight asymmetry. Although Egger’s regression test did not reach statistical significance (p = 0.2916), this result should be interpreted with caution due to limited statistical power given the small number of studies (n<10).**Supplement figure S3.** Forest plot comparing adverse events between ESG and IGB. Pooled analysis of four studies showed a non-significant trend favoring ESG (OR: 0.422; 95% CI: 0.062–2.884; *p* = 0.379), with substantial heterogeneity (I² = 78.38%). Adverse events in the IGB group commonly involved nausea, vomiting, and early device removal, while ESG-related events were less frequent and primarily included gastrointestinal bleeding and fluid collection.

**Supplementary Figure S4.** Sensitivity meta-regression of the treatment effect size (%TBWL difference) on the overall mean baseline BMI across both study arms. This analysis models the therapeutic advantage of ESG over IGB as a function of the aggregate obesity severity of the included study populations. Each circle represents an individual study, with the size corresponding to the study's weight in the random-effects model. A significant positive association was observed (coefficient = 0.0705, p = 0.0031), confirming that the superior weight loss efficacy of ESG is more pronounced in studies involving cohorts with higher overall baseline BMI. This study-level association supports the robustness of the primary findings independent of the inter-group baseline BMI difference.

 **Supplementary Figure S5.** Sensitivity meta-regression of the treatment effect size (%TBWL difference) on the mean baseline BMI of the ESG cohort.

To further address potential selection bias and the ecological fallacy, this sensitivity analysis utilizes the mean baseline BMI specific to the ESG cohort as the moderator. The results demonstrate a highly significant study-level association (coefficient = 0.0695, p = 0.0018). This finding indicates that the treatment benefit of ESG over fluid-filled balloons scales with the severity of obesity in the treated population, providing evidence that ESG is particularly effective for patients in higher BMI categories. These data reinforce the stability of the meta-regression outcomes across different model specifications.


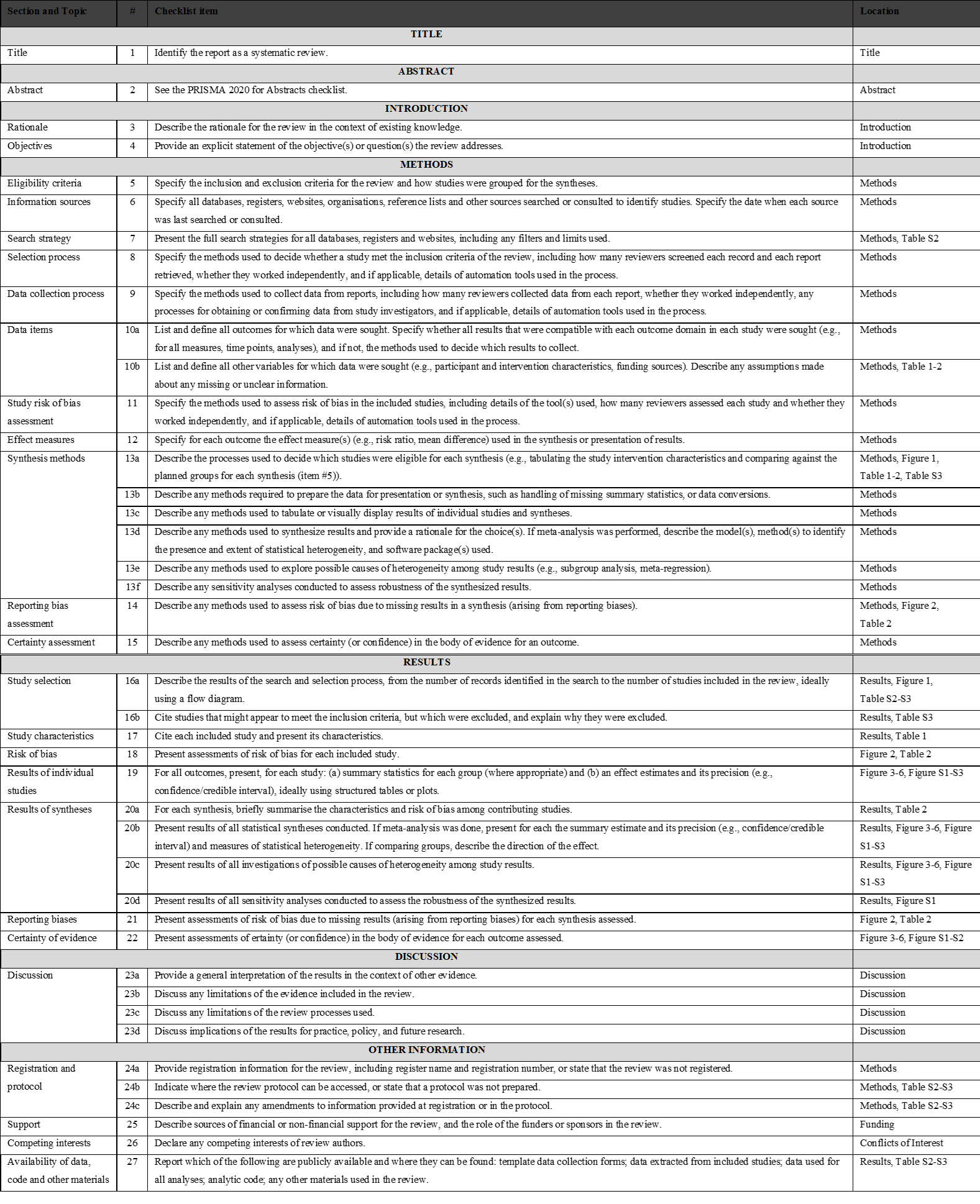


Supplementary Table S1 **PRISMA Checklist**

Supplement Table S2 **Keywords and search results in different databases**

Supplement Table S3. **Excluded studies and reasons**

Supplement Table S4. **Summary of Findings table based on GRADE assessment for ESG versus IGB in adults with obesity**

| First Author  Year | Adjustment Method | Confounders Adjusted  (ROBINS-I Style) | Confounding Risk |
| --- | --- | --- | --- |
| Fayad  2019 | Multivariate Regression | Age, Sex, and Baseline BMI ; both groups received identical nutritional protocols | Moderate |
| Gudur  2023 | Propensity Score Matching (1:1) | Age, Sex, Race, Baseline BMI, and multiple comorbidities | Low |
| Petriczko  2022 | Comparative Cohort | Identical post-procedure nutritional and behavioral protocols | Moderate-High |
| Limas  2024 | Comparative Cohort | Same multidisciplinary team and protocol for both groups | Moderate-High |
| Lopez-Nava  2019 | Multivariate Regression | Baseline BMI, Follow-up adherence ; identical standardized MDT protocols | Moderate |
| Rapaka  2022 | Prospective Comparative | Primary focus on gastric physiology; minimal baseline adjustment for weight outcomes | High |

Supplement Table S5. **Evaluation of potential confounding and adjustment strategies across included studies**.
